# Occurrences and Functions of Ly6C^hi^ and Ly6C^lo^ Macrophages in Health and Disease

**DOI:** 10.3389/fimmu.2022.901672

**Published:** 2022-05-30

**Authors:** Yuan-hui Li, Yu Zhang, Gang Pan, Li-xin Xiang, Ding-cun Luo, Jian-zhong Shao

**Affiliations:** ^1^ Department of Oncological Surgery, Affiliated Hangzhou First People’s Hospital, Zhejiang University School of Medicine, Hangzhou, China; ^2^ College of Life Sciences, Key Laboratory for Cell and Gene Engineering of Zhejiang Province, Zhejiang University, Hangzhou, China; ^3^ Laboratory for Marine Biology and Biotechnology, Qingdao National Laboratory for Marine Science and Technology, Qingdao, China

**Keywords:** Ly6C^hi^ and Ly6C^lo^ macrophages, differentiation, inflammation, autoimmune disease, cancer

## Abstract

Macrophages originating from the yolk sac or bone marrow play essential roles in tissue homeostasis and disease. Bone marrow-derived monocytes differentiate into Ly6C^hi^ and Ly6C^lo^ macrophages according to the differential expression of the surface marker protein Ly6C. Ly6C^hi^ and Ly6C^lo^ cells possess diverse functions and transcriptional profiles and can accelerate the disease process or support tissue repair and reconstruction. In this review, we discuss the basic biology of Ly6C^hi^ and Ly6C^lo^ macrophages, including their origin, differentiation, and phenotypic switching, and the diverse functions of Ly6C^hi^ and Ly6C^lo^ macrophages in homeostasis and disease, including in injury, chronic inflammation, wound repair, autoimmune disease, and cancer. Furthermore, we clarify the differences between Ly6C^hi^ and Ly6C^lo^ macrophages and their connections with traditional M1 and M2 macrophages. We also summarize the limitations and perspectives for Ly6C^hi^ and Ly6C^lo^ macrophages. Overall, continued efforts to understand these cells may provide therapeutic approaches for disease treatment.

## Introduction

Macrophages contribute to homeostasis and disease through their extensive tissue distribution, functional diversity and plasticity. Tissue-resident macrophages (TRMs) arise from two sources: embryonic precursors and circulating monocytes (1–3). Embryonic macrophages contribute to self-maintenance, tissue remodeling and genotoxic stress resistance (4, 5), whereas bone marrow monocyte-derived macrophages act as short-lived effector cells contributing to various physiological activities, such as atherosclerosis and fibrosis (6). Conventionally, macrophages with different functions are described as M1 and M2 macrophage subsets (7). The M1 macrophages are known as classically activated macrophages, which contribute to primary host defense against pathogens (8). The M2 macrophages are known as alternatively activated macrophages, which heal tissue injury or damage caused by M1 macrophages and are involved in stimulating antibody production in adaptive humoral immunity (9). In addition, an increasing number of tumor-associated macrophages (TAMs) have been identified, which execute diverse functions such as suppression of antitumor immunity (10). The concept of M1 and M2 macrophage subsets is mainly derived from the *in vitro* polarization inducing assays, hence the M1 and M2 classifications are more suitable to describe the activation state of macrophages *in vitro*.

During the recent decade, the new classification of Ly6C^hi^ and Ly6C^lo^ macrophages has been widely applied to investigate monocyte-derived macrophages and to depict the precise state of macrophages in an intricate internal microenvironment (11). This classification system represents two different macrophage populations that are distinct in phenotype, function and even origin (12–14). Ly6C is a glycoprotein that is expressed on macrophage/dendritic cell precursors during mid-stage development. Differential Ly6C expression can identify functionally distinct macrophage populations in the steady state or disease (15, 16). In mice, the circulating monocytes derived from bone marrow are composed of at least Ly6C^hi^ and Ly6C^lo^ subsets. The CX3CR1^mid^CCR2^+^Ly6C^hi^ and CX3CR1^hi^CCR2^-^Ly6C^lo^ phenotypes are functional equivalent with CD14^hi^CD16^lo^ and CD14^lo^CD16^hi^ phenotypes in humans (17, 18). Recently, emerging studies have shown that the continuum of macrophage phenotypes, not the two circumscribed profiles originally proposed, plays important roles in various diseases, including kidney injury (19), liver fibrosis (13), rheumatoid arthritis (20), and breast cancer (21). Here, we review the new classification and perspectives in monocyte-derived macrophage research, including the origin, heterogeneity, conversion, and function of Ly6C^hi^ and Ly6C^lo^ macrophages.

## Basic Biology of LY6C^HI^ and LY6C^LO^ Macrophages

### Origin, Development, and Functional Heterogeneity

For half a century or more, the prevailing doctrine for tissue macrophages has been that these cells originate from circulating monocytes (22). Recently, it has become obvious that most tissue macrophages originate during embryonic development (23). TRM populations are mainly contributed by yolk sac (YS)-derived macrophages, erythro-myeloid progenitors (EMPs), and fetal hematopoietic stem cells (HSCs) (24, 25). In mice, yolk sac- or fetal liver-derived macrophages are located in different adult tissues, including the brain, epidermis and kidneys, and contribute to tissue homeostasis independent of bone marrow-derived monocytic precursors (5, 24, 25). Embryonic- derived and monocytes-derived subsets contribute to macrophage in adult tissues, including the gut and dermis (26–29). In addition, in the heart and pancreas, the macrophage population is a mixed population of yolk sac-derived macrophages, fetal liver-derived monocytes and bone marrow-derived monocytes (5).

Although tissues are populated with fetal macrophages, monocyte-derived macrophages might replace TRMs to a greater or lesser extent. Monocyte-derived macrophages are classified as CD11B^hi^F4/80^hi^Ly6C^hi^ macrophages (namely, Ly6C^hi^ macrophages) and CD11B^hi^ F4/80^hi^Ly6C^lo^ macrophages (namely, Ly6C^lo^ macrophages) based on the expression of Ly6C, a cell-surface glycoprotein (14, 19). Ly6C^hi^ macrophages develop from recruited classical CCR2^+^CX3XR1^lo^Ly6C^hi^ monocytes (analogous to human CD14^+^CD16^-^ monocytes) during inflammation and are then converted into Ly6C^lo^ macrophages. With the development of techniques such as single-cell sequencing and mass cytometry, new dimensions of the richness and heterogeneity of macrophages have been mapped. According to the latest single-cell analysis studies, four subpopulations of Ly6C^hi^ inflammatory macrophages have been found to be present in kidney injury through relatively meticulous research (3). Here, we focus on the two most unlike subsets, Ly6C^hi^ and Ly6C^lo^ macrophages, to reveal the phenotypic and functional differences between them.

The Ly6C^hi^ and Ly6C^lo^ subsets exhibit functional heterogeneity, which is indicated by the high diversity in cell-surface marker, cytokine release and transcriptional profiles (14, 30–32). Indeed, Ly6C^hi^ macrophages derived from circulating Ly6C^hi^ monocytes are more enriched in the acute inflammatory response and show a proinflammatory ability (3). They exert proinflammatory and profibrotic functions mediated through various inflammatory and secreted factors, including tumor necrosis factor (TNF), interleukin (IL)-1β, and transforming growth factor (TGF)-β (33, 34). In contrast, Ly6C^lo^ macrophages attract wide attention for their protective roles in wound healing, anti-inflammatory processes, and antifibrotic processes (14, 35, 36). Ly6C^lo^ macrophages play diverse roles in maintaining the stability of the endothelium, regulating vasculogenesis, and transporting ions (3). They supposedly derived from Ly6C^lo^ monocytes which are known to patrol endothelial cell of the blood vasculature (37). The detailed functions of the two subsets are discussed below (**Table 1**).

**Table 1:** The role of Ly6C^hi/lo^ macrophages in disease.

Disease	Ly6C^hi/lo^ subset	Tissue/organ/cell line	Channel	Conclusion	Reference
Liver injury	Ly6C^hi^ M	Liver	CCL2, TNF, IL-1	Detrimental	(32, 39)
Kidney injury	Ly6C^hi^ M	Kidney	TLR4-dependent inflammatory signaling pathways	Detrimental	(3)
Kidney injury	Ly6C^lo^ M	Kidney	Phagocytosis, regulation of angiogenesis	Protective	(3)
Kidney injury	Ly6C^lo^ M	Kidney	CCL-17, CCL-22, IGF-1, and PDGF-β	Detrimental	(15)
Colitis	Ly6C^hi^ M	intestine	IL-1β, TNF	Detrimental	(66)
Skeletal muscle injury	Ly6C^lo^ M	Skeletal muscle	Resolvin D2	Protective	(36)
Sciatic nerve injury	Ly6C^lo^ M	Sciatic nerve	Efferocytosis	Protective	(47)
Myocardial infarction	Ly6C^lo^ M	Heart	TGF-β	Protective	(48)
Liver fibrosis	Ly6C^hi^ M	Liver	IL-1β, TNF,TGF-β	Detrimental	(14)
Liver fibrosis	Ly6C^lo^ M	Liver	MMP9, MMP12, MMP13, HGF, IGF, Mertk, Trem2	Protective	(14)
Lung fibrosis	Ly6C^hi^ M	Lung	TGF-β	Detrimental	(68)
RA	Ly6C^hi^ M	Joint	IL-1β, IL-6, IL-8, and TNF-α	Detrimental	(20)
RA	Ly6C^lo^ M	Joint	Differentiate into inflammatory macrophages (M1)	Detrimental	(73)
RA	Ly6C^lo^ M	Joint	Through the mobilization of Treg cells	Protective	(74)
SLE	Ly6C^hi^ M	Kidney	CSF-1	Detrimental	(76)
SLE	Ly6C^lo^ M	Kidney	MMPs	Protective	(77)
MS	Ly6C^hi^ M	Central nervous system	TNF-α, iNOS, p40, p19, IL-6	Detrimental	(78)
Breast cancer	Ly6C^hi^ M	Breast cancer	CCL2-CCR2 axes	Detrimental	(84)
Leukemia	Ly6C^hi^ M	Bone marrow	TNF-α, IL-1β, IFN-γ, CCL8–CCR1/CCR2 axes, CCL9/10–CCR1 axes	Detrimental	(88, 89)
Breast cancer	Ly6C^lo^ M	Mammary adenocarcinoma cell line	iNOS, Cox2, IL-1β, IL-6	Detrimental	(90)
Breast cancer	Ly6C^lo^ M	Breast cancer	A-FABP	Detrimental	(21)
Lung metastasis	Ly6C^lo^ M	Lung	IL-6	Detrimental	(92)

### Conversion of Ly6C^hi^ Macrophages Into Ly6C^lo^ Macrophages

**Figure 1:**
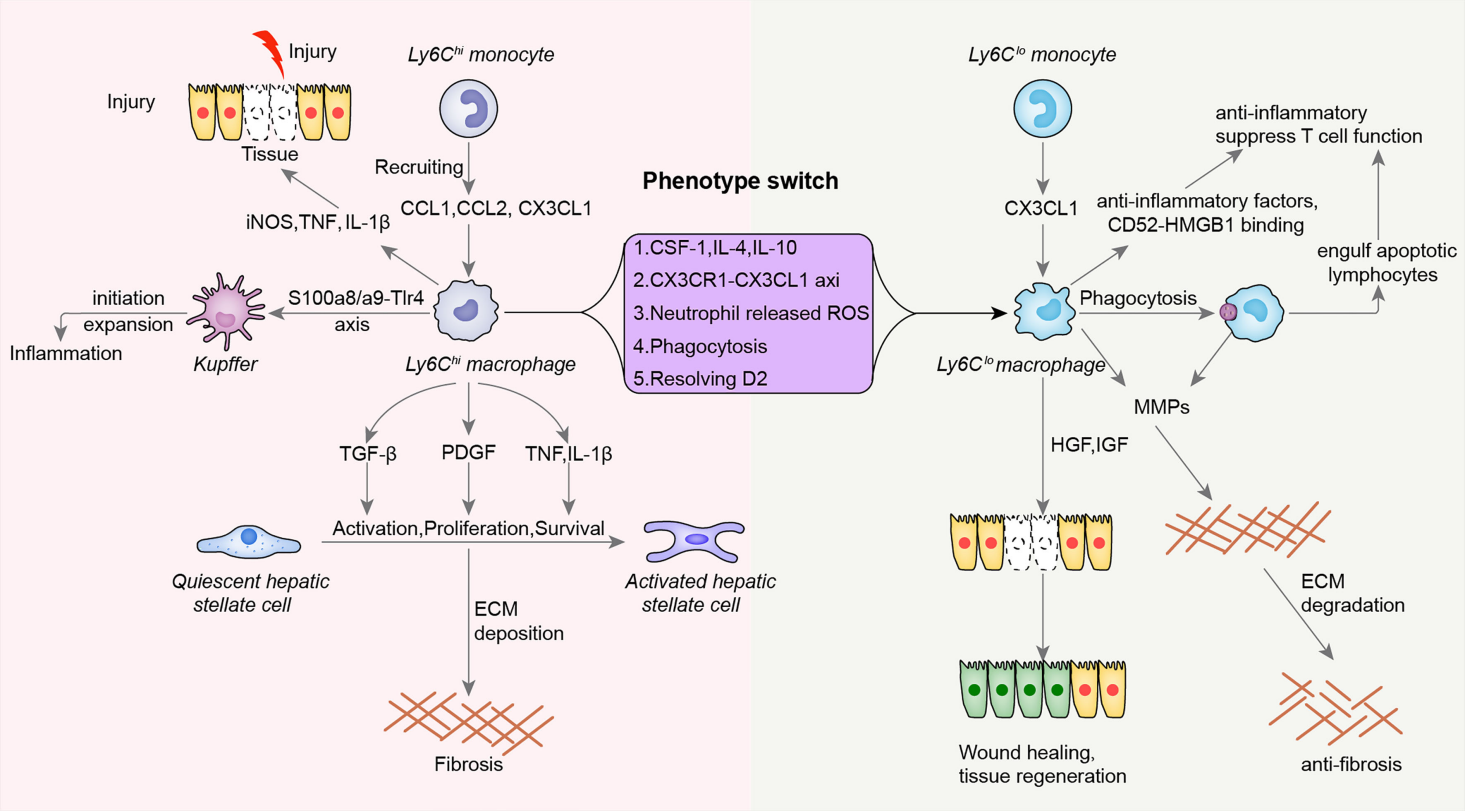
The distinct functions of Ly6C^hi^ and Ly6C^lo^ macrophages in injury, chronic inflammation and wound repair. During the initial stage of inflammation, Ly6C^hi^ monocytes are recruited to the injury site *via* cytokines, including CC-chemokine ligand 1 (CCL1), CC-chemokine ligand 2 (CCL2), and fractalkine (CX3CL1), wherein they develop into Ly6C^hi^ macrophages. These cells express proinflammatory mediators, such as nitric oxide (NO), tumor necrosis factor (TNF) and interleukin-1β (IL-1β), which exacerbate tissue injury. These Ly6C^hi^ cells interact with kidney-resident macrophages through the S100a8/a9-Tlr4 axis, initiating and amplifying the inflammatory response. During fibrosis, Ly6C^hi^ cells promote this process through the effects of transforming growth factor-β (TGFβ) on quiescent hepatic stellate cell activation, platelet-derived growth factor (PDGF) on myofibroblast proliferation and TNF and IL-1β on activated hepatic stellate cells. Ly6C^lo^ macrophages accumulate *via* the recruitment of Ly6C^lo^ monocytes or phenotype switching from Ly6C^hi^ macrophages. Proresolution macrophages inhibit the inflammatory response and T-cell function through anti-inflammatory factors and CD52-HMGB1 binding. They also engulf apoptotic T cells, which play anti-inflammatory roles. During the phagocytic process, Ly6C^lo^ cells produce matrix metalloproteinases (MMPs), such as MMP9, MMP12, and MMP13, accelerating extracellular matrix (ECM) degradation and inhibiting fibrosis. During tissue repair and reconstruction, Ly6C^lo^ cells secrete hepatocyte growth factor (HGF) and insulin-like growth factor (IGF) to promote wound healing and tissue regeneration. Regarding the mechanism underlying phenotypic switching, various factors and processes, including cytokines (CSF-1, IL-4, and IL-10), the CX3CR1-CX3CL1 axis, neutrophil-released ROS, phagocytosis, and Resolving D2, have been explored.

Conversion of Ly6C^hi^ macrophages into Ly6C^lo^ macrophages through phenotypic switching is an important source of tissue macrophages (13, 14, 33), but the precise regulatory mechanism underlying this process is still unclear. However, studies have revealed the signals driving Ly6C^hi^/Ly6C^lo^ monocyte conversion and its molecular bases (55, 56). For example, it was proposed that the conversion of Ly6C^hi^ monocytes into Ly6C^lo^ monocytes might be a functional transition caused by loss of microenvironmental signals that sustain the expression of genes specific to Ly6C^hi^ cells rather than a true developmental, terminal differentiation program (57, 58). The transcription factors Nr4a1 and Cebpβ were reported to be master regulators that promote the conversion of Ly6C^hi^ monocytes into Ly6C^lo^ monocytes (56, 59, 60). Both Nr4a1^−/−^ mice and Cebpβ^−/−^ mice lack Ly6C^lo^ monocytes (59, 61). In early studies, macrophage colony-stimulating factor 1 (CSF-1) was shown to promote the maturation of macrophages from bone marrow-derived macrophage precursors accompanied by a rapid decrease in Ly6C expression, which indicated the significance of CSF-1 in phenotypic switching (62, 63). Apart from this, IL-4 and IL-10 were found to have the ability to promote liver-derived Ly6C^hi^ macrophage conversion into Ly6C^lo^ macrophages, and a synergistic effect was observed between these two cytokines (13). Resolving D2, a specialized proresolving lipid mediator, significantly improves muscle regeneration by promoting Ly6C^hi^/Ly6C^lo^ macrophage conversion (36). Phagocytosis by Ly6C^hi^ macrophages fosters Ly6C^hi^/Ly6C^lo^ macrophage conversion in the fibrotic liver through liposomal stimulation (14). In addition, the CX3CR1-CX3CL1 signaling axis indirectly regulates the phenotypic switch between Ly6C^hi/lo^ macrophages (38, 40, 43, 64). Since Ly6C^lo^ macrophages are beneficial for hepatic fibrosis resolution, the number of Ly6C^hi^ macrophages was significantly increased in CX3CR1^-/-^ mice, followed by chronic inflammation and increased hepatic fibrosis (40). Moreover, neutrophils are involved in the Ly6C^hi^/Ly6C^lo^ macrophage switch by expressing reactive oxygen species (ROS) to orchestrate liver repair (41) (**Figure 1**). Although the master regulators involved in Ly6C^hi^/Ly6C^lo^ macrophage conversion are not fully understood, many investigations are contributing to the answer.

## Ly6C^HI^ and Ly6C^LO^ Macrophages in Homeostasis and Pathology

### Ly6C^hi^ and Ly6C^lo^ Macrophages in Homeostasis

In the steady state, tissue-resident macrophages, such as microglia, Langerhans cells, and Kupffer cells, exhibit a F4/80^hi^Ly6C^lo^ phenotype. These tissue-resident macrophages play fundamental homeostatic roles in the clearance of apoptotic cells and participate in tissue immune surveillance (42). They maintain themselves locally and independently of circulating precursors. For instance, the most important cardiac macrophages in the steady state are F4/80^hi^Ly6C^lo^MHCII^hi^ and F4/80^hi^Ly6C^lo^MHCII^lo^ subsets. These subsets exist independently of bone marrow-derived monocytes and are renewed through *in situ* proliferation. They perform more antigen sampling and efferocytosis than infiltrating Ly6C^hi^ macrophages (27). In contrast, Ly6C^hi^ macrophages are rarely involved in tissue homeostasis. Classical Ly6C^hi^ monocytes do not enter tissues on a large scale, and they intend to switch to a Ly6C^lo^ phenotype with time of residency. Ly6C^hi^ monocytes remain in an undifferentiated state instead of becoming committed macrophages or DCs, which is different from the differentiation of Ly6C^hi^ monocytes into macrophages or DCs during inflammation (23). However, when homeostasis is disrupted, bone marrow-derived Ly6C^hi^ monocytes are recruited to the site of inflammation.

### Ly6C^hi^/Ly6C^lo^ Macrophages in Injury, Chronic Inflammation and Wound Repair

The proinflammatory and profibrotic roles of Ly6C^hi^ macrophages has been reported in various diseases, among which liver injury and fibrosis are typical models used to investigate the function of the Ly6C^hi^ subset. During acute liver injury and chronic liver diseases such as liver fibrosis, CCR2^+^Ly6C^hi^ monocytes are recruited to the liver in a manner dependent on the CCL2/CCR2 or CCL1/CCR8 chemokine-receptor interaction (39, 61). In the liver, these cells develop into infiltrating Ly6C^hi^ macrophages and exhibit a proinflammatory phenotype. Ly6C^hi^ macrophages express inflammatory genes, including inducible nitric oxide synthase (iNOS) and TNF, which aggravate the inflammatory response (65). In other inflammatory diseases, such as acute lung injury (19) colitis (66) and skin wound healing (28, 44, 67), the Ly6C^hi^ subset is the source of IL-1β and TNF, and Ly6C^hi^ macrophage-targeted therapies are useful for decreasing inflammation. Functionally, Ly6C^hi^ macrophages intensify the scarring occurring during liver fibrosis by promoting hepatic stellate cell (HSC) survival *via* IL-1β and TNF-induced NF-κB activation and TGF-β/PDGF-mediated HSC transdifferentiation and proliferation (45, 46). Inhibiting infiltrating Ly6C^hi^ monocytes in CCR2^-/-^ mice was shown to relieve liver fibrosis (39). Similarly, the profibrotic function of Ly6C^hi^ macrophages in lung and kidney fibrosis has been revealed (19, 43, 68). Mechanistically, Ly6C^hi^ macrophages with high S100a8 and S100a9 expression were found to have a strong interaction with kidney-resident macrophages through the S100a8/a9-Tlr4 axis, thereby initiating and amplifying the inflammatory response during kidney injury (3) (**Figure 1**).

In contrast to Ly6C^hi^ macrophages, Ly6C^lo^ macrophages play important roles in inhibiting inflammation, promoting wound healing, improving regeneration and decreasing fiber deposition during tissue injury and fibrosis (**Figure 1**). Taking liver fibrosis as an example, the restorative Ly6C^lo^ subset upregulates phagocytosis-related genes (*Fcrls*, *Cd5l*, *Mertk*, *Trem2*, and *Axl*), matrix degradation-related genes (*Mmp9*, *Mmp12*, and *Mmp13*), and growth factors (*Hgf*, *Igf1* and *Mif*), which facilitate fiber degradation, fibrosis resolution, and tissue protection (14). In sciatic nerve injury, inflammation-resolving Ly6C^lo^ macrophages derived from Ly6C^hi^ cells promote an anti-inflammatory milieu by efferocytosis (47). In skeletal muscle injury, the proresolving lipid mediator resolvin D2 increases Ly6C^lo^ macrophages and improves muscle regeneration (36). Ly6C^lo^ macrophages express genes closely related to the mitotic cell cycle and cell division and are involved in various biological processes, including defense reactions and responses to cytokine stimuli and viruses, after resolvin D2 treatment (36). In myocardial infarction, Ly6C^lo^ macrophages play crucial roles in postinfarct healing and optimal scar formation by secreting immunoregulatory factors, such as TGF-β (48). However, a destructive role for Ly6C^lo^ macrophages has also been reported. For example, bone marrow-derived Ly6C^lo^ macrophages worsen renal fibrosis by secreting various cytokines that promote the transdifferentiation of fibroblasts into myofibroblasts (69). This viewpoint is quite different from those in previous reports, and the debate on this needs to be resolved.

### Ly6C^hi^ and Ly6C^lo^ Macrophages in Autoimmune Disease

The detrimental functions of Ly6C^hi^ macrophages in autoimmune disease has been revealed in various reports. However, Ly6C^lo^ macrophages show harmful or beneficial functions in diverse pathological conditions and different autoimmune disease types (**Table 1**). Rheumatoid arthritis (RA) is a complex autoimmune disease influenced by both genetic and environmental factors (70). Macrophages and monocytes have been reported to play important roles in the pathophysiology of RA (71). Ly6C^hi^ macrophages have been reported to aggravate the progression of RA. Decreases in Ly6C^hi^ macrophage numbers and chemokines are favorable markers for clinical improvement with treatment (49). Infliximab was used to improve RA in human TNF transgenic (hTNF-Tg) mice, functioning mainly by inducing apoptosis in Ly6C^hi^ macrophages and inhibiting the recruitment of Ly6C^hi^ monocytes (49). Ly6C^lo^ macrophages are believed to have diverse functions in RA. Serum transfer-induced arthritis (STIA) mice are good model for RA studies (72, 73). Researchers revealed that Ly6C^lo^ monocytes were recruited to arthritic joints and developed into Ly6C^lo^MHC-II^+^ and Ly6C^lo^MHCII-macrophages; among these cells, Ly6C^lo^MHCII-macrophages drove the development of joint pathology (73). However, in contrast, Ly6C^lo^ monocytes developed into Ly6C^lo^ macrophages, which resembled anti-inflammatory M2 macrophages and contributed to reducing joint inflammation through the mobilization of regulatory T (Treg) cells (74).

Systemic lupus erythematosus (SLE) is a heterogeneous systemic rheumatic disease with profound effects on multiple organs (75). In a mouse model of lupus nephritis (MRL-Fas^lpr^ mice), CSF-1 shifted circulating Ly6C^hi^ monocytes toward inflammatory Ly6C^hi^ macrophages that induce apoptosis in tubular epithelial cells, damaging the kidneys (76). A similar study showed that the Ly6C^hi^ subset increased notably and secreted proinflammatory cytokines and chemokines during SLE (77). However, Ly6C^lo^ macrophages possess distinct functions in SLE. At nephritis onset, Ly6C^lo^ macrophages upregulate the cell-surface marker CD11b, acquire cathepsin and matrix metalloproteinase activity, and protect cells from death. However, these changes reverse after the induction of remission (77). Multiple sclerosis (MS) is a chronic autoimmune disease mediated by a complex interaction between autoreactive lymphocytes and myeloid cells in the central nervous system (CNS) and is the most common inflammatory neurological disease in young adults (78). Experimental autoimmune encephalomyelitis (EAE), characterized by immune cell infiltration of the CNS, is an ideal model for investigating MS (79). Various studies in EVE models have revealed that CCR2^+^Ly6C^hi^ monocytes are required for the initiation and progression of EVE (50, 51, 79). Ly6C^hi^ macrophages derived from Ly6C^hi^ monocytes are essential for the maintenance of chronic inflammation and the progression of EVE. Acetylcholine-producing natural killer (NK) cells were shown to be cytotoxic to Ly6C^hi^ cells in EVE, acting by inhibiting the production of proinflammatory cytokines and thereby attenuating CNS inflammation (80). Autoimmune (noninfectious) uveitis is a group of intraocular inflammatory diseases that target the neuroretina, and this disease can affect the CNS (81). In mice, experimental autoimmune uveitis (EAU) is a model of organ autoimmunity in the eye. By using this model, HSC-derived Ly6C^hi^ and Ly6C^lo^ macrophages with relatively high MHC-II expression were found to be associated with EAU through their antigen-presenting and CD4^+^ T cell-activating activities (52).

### Ly6C^hi^ and Ly6C^lo^ Macrophages in Cancer

#### Ly6C^hi^ Macrophages in Cancer

Ly6C^hi^ macrophages extensively enhance tumor initiation and malignant progression. They build an inflammatory microenvironment to promote tumor growth, invasion and metastasis. The roles of CCL2/CCR2 signaling and Ly6C^hi^ monocyte recruitment have been implicated as poor prognostic factors in multiple malignancies (53, 54, 82, 83). CCL2/CCR2 signaling was reported to foster metastasis and prolong the survival of tumor-bearing mice, and CCL2 expression and macrophage infiltration are correlated with a poor prognosis and metastatic disease in human breast cancer (84). An anti-CCL2 antibody was found to inhibit the infiltration of Ly6C^hi^ monocytes and tumor metastasis (84). However, CCR2-independent pathway also influenced recruitment under noninflammatory conditions (85). CSF-1 signaling has been reported to determine monocyte recruitment and differentiation in the tumor microenvironment. CSF1R signaling blockade impairs the extravasation of tumor-infiltrating Ly6C^hi^ monocytes (86). In addition, Ly6C^hi^ macrophages are closely connected with immune resistance to ablative radiotherapy in pancreatic ductal adenocarcinoma, as depletion of this subset delays tumor growth after radiotherapy (87). In leukemic mice, an increase in monocyte-derived Ly6C^hi^ leukemia-associated macrophages (LAMs) was detected in extramedullary tissue. Ly6C^hi^ LAMs differ from TAMs in their gene expression profile and activation phenotype. They actively express TNF-α and IL-1β, which contribute to sterile inflammation. Ly6C^hi^ LAMs have high migratory and phagocytotic potentials and promote the extramedullary distribution of leukemia cells (88, 89).

#### Ly6C^lo^ Macrophages in Cancer

The Ly6C^lo^ macrophages also demonstrate detrimental roles during tumor progression. They promote angiogenesis, exert immunosuppressive effect, and are associated with poor prognosis. The monocyte pool in tumors almost exclusively consists of Ly6C^hi^CX3CR1^lo^ monocytes, which renew TAM subsets (90). These inflammatory monocytes undergo rapid differentiation into TAMs and, in doing so, lose Ly6C expression (87). TAMs are distinguished as normoxic M1-like Ly6C^lo^MHC-II^hi^ TAMs and hypoxic M2-like Ly6C^lo^MHC-II^lo^ TAMs (90, 91). Among TAMs, the Ly6C^lo^MHC-II^lo^ subset was found to be the main population involved in tumor growth, invasion and metastasis in the mammary adenocarcinoma TS/A model. Although the Ly6C^lo^MHC-II^lo^ subset exhibits weak antigen presentation, they promote angiogenesis and suppress T-cell proliferation (90). The differentiation of Ly6C^hi^ monocytes into the Ly6C^lo^MHC-II^lo^ subset is facilitated by CSF1R signaling (86). In breast cancer, the expression of adipocyte/macrophage fatty acid binding protein (A-FABP) in TAMs, especially the Ly6C^lo^MHC-II^lo^ subset, was shown to facilitate tumor progression. A-FABP expression in the Ly6C^lo^MHC-II^lo^ subset promoted protumor *IL-6/STAT3* signaling through regulation of the *NF-κB/miR-29b* pathway (21). During lung metastasis, tumor cell-released microparticles (T-MPs) foster the recruitment of inflammatory monocytes, and these cells mature into Ly6C^lo^ macrophages. Ly6C^lo^ cells not only produce IL-6 but also trigger fibrin deposition, facilitating the growth and survival of tumor-repopulating cells, thus setting the stage for lung metastasis (92). As described above, Ly6C^lo^ TAMs are associated with a poor prognosis and tumor progression in multiple cancers. However, a proinflammatory Ly6C^lo^MHC-II^+^ macrophage subset was confirmed to promote responsiveness to PD-L1 blockade instead of resistance; thus, this subset may have a host-protective role in immune checkpoint blockade therapies (93).

## Correlation Between Ly6C^HI/LO^ Macrophages and the M1/M2 Paradigm

Macrophages with distinct functions are traditionally classified as M1 macrophages (classically activated macrophages) and M2 macrophages (alternatively activated macrophages). Cells with the M1 phenotype participate in host defense against pathogens and antitumor immunity. However, those with the M2 phenotype possess anti-inflammatory function and facilitate wound healing and tumor progression (11). Strictly, M1 and M2 macrophages represent only the states polarized by IFN-γ/LPS and IL-4/IL-13 *in vitro*, respectively (94). This taxonomic lineage clearly defines the two extreme types of the macrophage spectrum, which is especially beneficial for *in vitro* research. However, in a complex microenvironment *in vivo*, such as that in CCL_4_-induced liver fibrosis (14) or chronic alcoholic liver injury (95), macrophages can exist along a continuous spectrum, and the simple M1/M2 paradigm cannot describe the state of macrophages. Therefore, researchers have begun to focus on the Ly6C^hi/lo^ phenotype outside the M1/M2 classification and depict various roles in homeostasis and pathology according to this classification system. Ly6C^hi/lo^ and M1/M2 macrophages have overlap in the gene expression profile. Ly6C^hi^ macrophages express some signature M1 markers, including TNF, iNOS and IFN-γ, and M2 markers, including Chi3l3, TGF-β and IL-10. Ly6C^lo^ macrophages upregulate traditional M1 genes, such as CD16 and CD32, and express some M2-specific markers, including CD206 and CD301 (14, 96). Therefore, when the activation state of macrophages *in vivo* is described, these cells can be defined more accurately through the combination of macrophage origin, surface markers and factors inducing the macrophage activation state.

## Current Research Gaps and Future Perspectives

In summary, macrophages are a key innate immune cell subset that plays various roles in multiple biological processes. The conventional M1/M2 paradigm is widely applied to describe the state of macrophages. Owing to the limitations of the M1/M2 paradigm, the Ly6C^hi/lo^ classification is increasingly used to describe cells involved in various diseases because this precise depiction is based on cell origin, stimuli, and identification markers (94). Here, we summarize the indispensable functions of Ly6C^hi^ and Ly6C^lo^ macrophages in homeostasis and pathology. Recent study highlighted the importance of tissue niches (blood vessels and nerves) to the two subsets (97). After blood monocytes recruitment and differentiation, the two distinct subsets preferentially reside within different, but conserved, subtissular niches located adjacent to either nerve fibers (Ly6C^lo^MHCII^hi^) or blood vessels (Ly6C^hi^MHCII^lo^), which demonstrate conserved niche-dependent functional programming (97). In fact, whether these Ly6C^hi^ and Ly6C^lo^ macrophage subsets interact with their respective surroundings in function and metabolism needs to be further explored. New genetic tools promote the disclosure of macrophage heterogeneity. Recently, Kim et al. established a binary transgenic split Cre system that allows differential targeting and translatome analysis of CNS border-associated macrophages (98). Genetics-based RiboTag translatome profiling can be a valuable and complementary addition to single cell transcriptomics and can be widely applied in the future.

## Author Contributions

Y-hL and J-zS searched the literature and wrote the manuscript. D-cL prepared the figures. L-xX, YZ, and GP carefully checked the manuscript and helped to improve paragraphs. All authors contributed to the article and approved the submitted version.

## Funding

This work was supported by grants from Stem Cell and Translational Research, the National Key Research and Development Program of China (2016YFA0101001, 2018YFD0900503, 2018YFD0900505), the National Natural Science Foundation of China (32173003, 31630083).

## Conflict of Interest

The authors declare that the research was conducted in the absence of any commercial or financial relationships that could be construed as a potential conflict of interest.

## Publisher’s Note

All claims expressed in this article are solely those of the authors and do not necessarily represent those of their affiliated organizations, or those of the publisher, the editors and the reviewers. Any product that may be evaluated in this article, or claim that may be made by its manufacturer, is not guaranteed or endorsed by the publisher.
